# Coarctation Stenting in a Rare Case with Congenitally Corrected Transposition of the Great Arteries and the Bicuspid Aortic Valve

**Published:** 2020-01

**Authors:** Zahra Khajali, Majid Maleki, Bahram Mohebbi, Maryam Aliramezany

**Affiliations:** 1 *Department of Cardiovascular Medicine, Shaheed Rajaei Cardiovascular Medical and Research Center, Iran University of Medical Sciences, Tehran, Iran.*; 2 *Cardiovascular Research Center, Institute of Basic and Clinical Physiology Sciences, Kerman University of Medical Sciences, Kerman, Iran.*

**Keywords:** *Transposition of great vessels coarctation*, *Bicuspid aortic valve*, *Aortic coarctation*, *Surgical procedures, operative*

## Abstract

Congenitally corrected transposition of the great arteries (cc-TGA) is a rare congenital abnormality that occurs in 1 per 33000 live births. This abnormality comprises nearly 0.05% of all congenital heart defects, with at least 90% of cc-TGA patients having associated cardiac defects; some of these associated defects are, however, very rare. In this case report, we describe a 22-year-old man who referred to our hospital for the evaluation of hypertension and cardiac murmurs. Via echocardiography and catheterization, the patient was finally diagnosed with cc-TGA, bicuspid aortic valve, and coarctation of the aorta. He underwent successful percutaneous transarterial coarctoplasty without any complications at early and 6 months’ follow-up visits.

## Introduction

Congenitally corrected transposition of the great arteries (cc-TGA) is a rare congenital abnormality that occurs in 1 per 33000 live births, accounting for nearly 0.05% of all congenital heart defects. Leftward looping (L-loop), rather than the normal rightward looping (D-loop), of the primitive cardiac tube leads to this malformation.^1^ Although the familial recurrence of heart defects has been reported in patients with cc-TGA, the true etiology of the problem has yet to be discovered.^2^ In this anomaly, different cardiac associated anomalies have already been reported; nonetheless, there is a dearth of data on coarctation and bicuspid aortic valve. In this report, we describe the results of chest X-ray, echocardiography, and angiography of this association.

## Case Report

A 22-year-old man with a history of hypertension and cardiac murmurs identified in infancy referred to our adult congenital cardiac disease department for the evaluation and management of secondary hypertension.

A loud pansystolic murmur audible through the precordium and between the scapulae was identified during the clinical examination. Additionally detected was hypertension, with an ankle-arm index <1 (pressure in the right arm=162/75 mmHg and pressure in the right leg=129/76 mmHg) and no substantial decrease in the femoral pulses. All these findings were in favor of coarctation of the aorta.

All the other tests performed to investigate the other underlying causes of secondary hypertension were normal. In the initial investigation, the anteroposterior view of chest X-ray showed the straightening of the upper part of the left cardiac border (secondary to the parallel transposition of the great artery), the absence of the main pulmonary trunk, and normal pulmonary vascularization. Electrocardiography revealed the sinus rhythm, signs of right atrial enlargement, Q waves in leads III and aVF, the mean cardiac axis in the frontal plane between 0^º^ and −30^º^, and a poor R progression in the precordial leads, all denoting the probability of cc-TGA.

**Figure 1:**
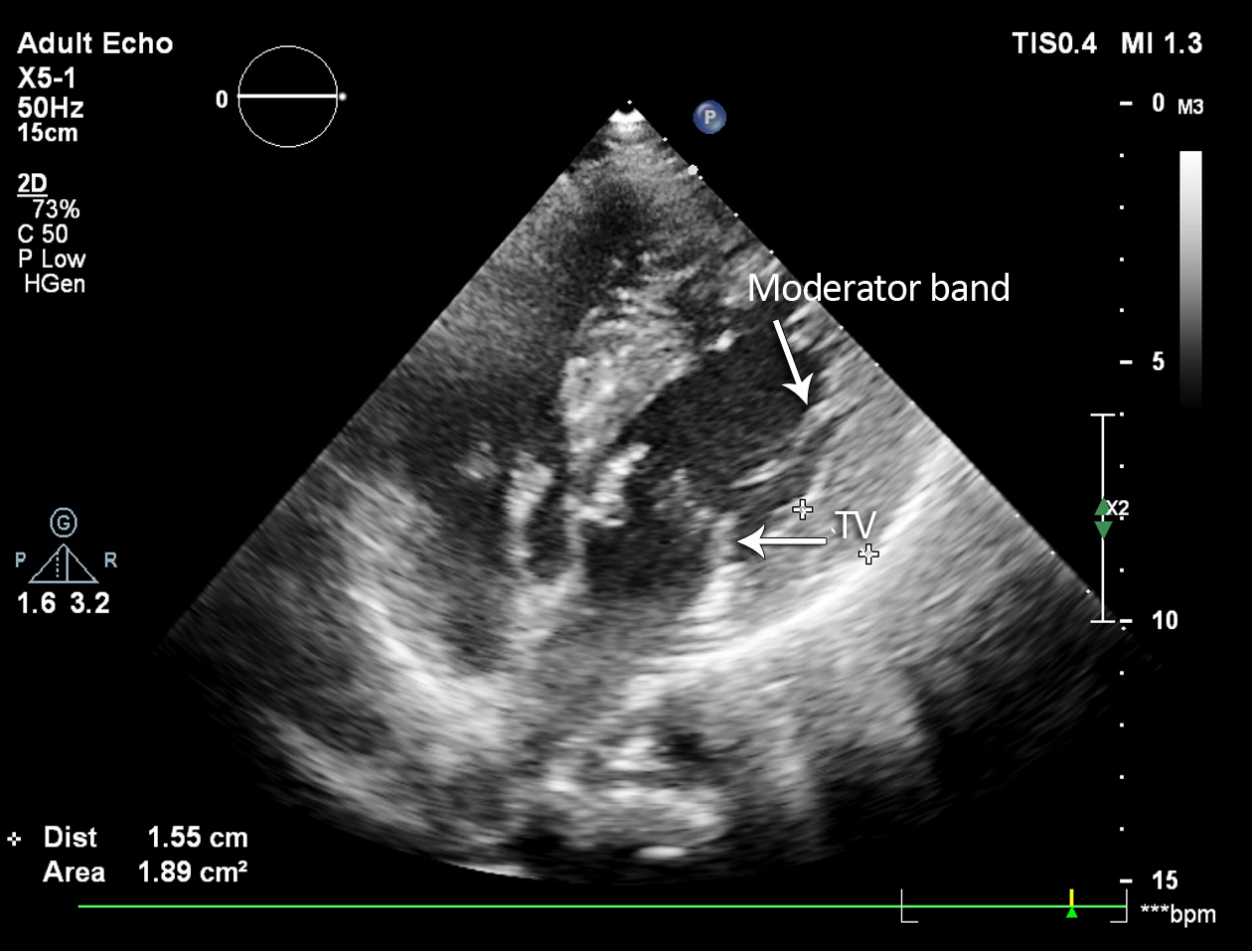
Two-dimensional 4-chamber view in transthoracic echocardiography, showing that the tricuspid valve (TV) is inserted close to the apex when compared with the mitral valve. Additionally, a markedly hypertrophic moderator band is seen in the left-sided ventricle.

**Figure 2:**
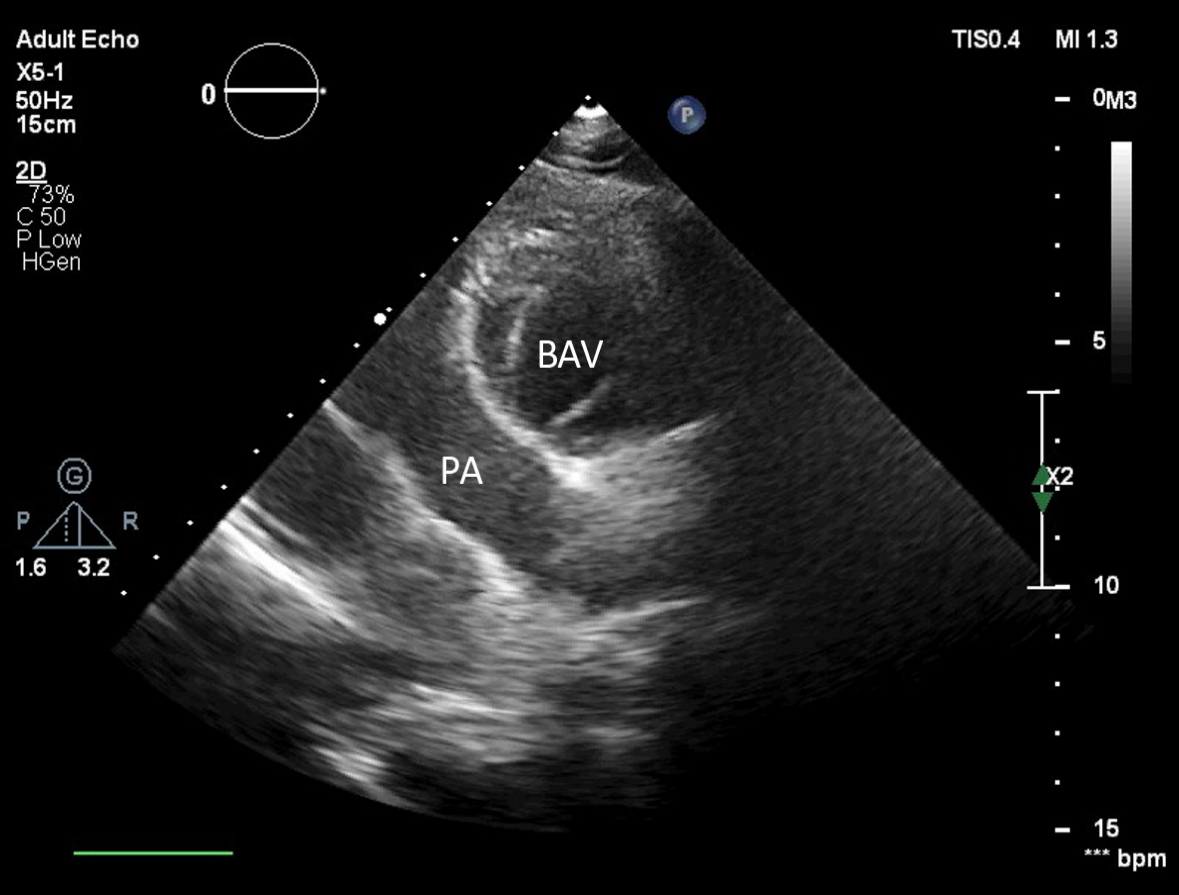
Two-dimensional transthoracic echocardiography in the short-axis parasternal view, showing a bicuspid aortic valve (BAV) and the anterior position of the aorta to the pulmonary artery (PA)

Transthoracic echocardiography was performed; and as is shown in Figure 1, atrioventricular (AV) and ventriculoarterial discordance was patently visible in the 4-chamber view. The aortic valve was bicuspid without a significant pressure gradient (Figure 2). Aortic coarctation distal to the left subclavian artery was seen through the suprasternal window, with a maximum Doppler gradient of 60 mmHg. Additionally, mild tricuspid regurgitation was noted, with no intracardiac shunts. 

**Figure 3:**
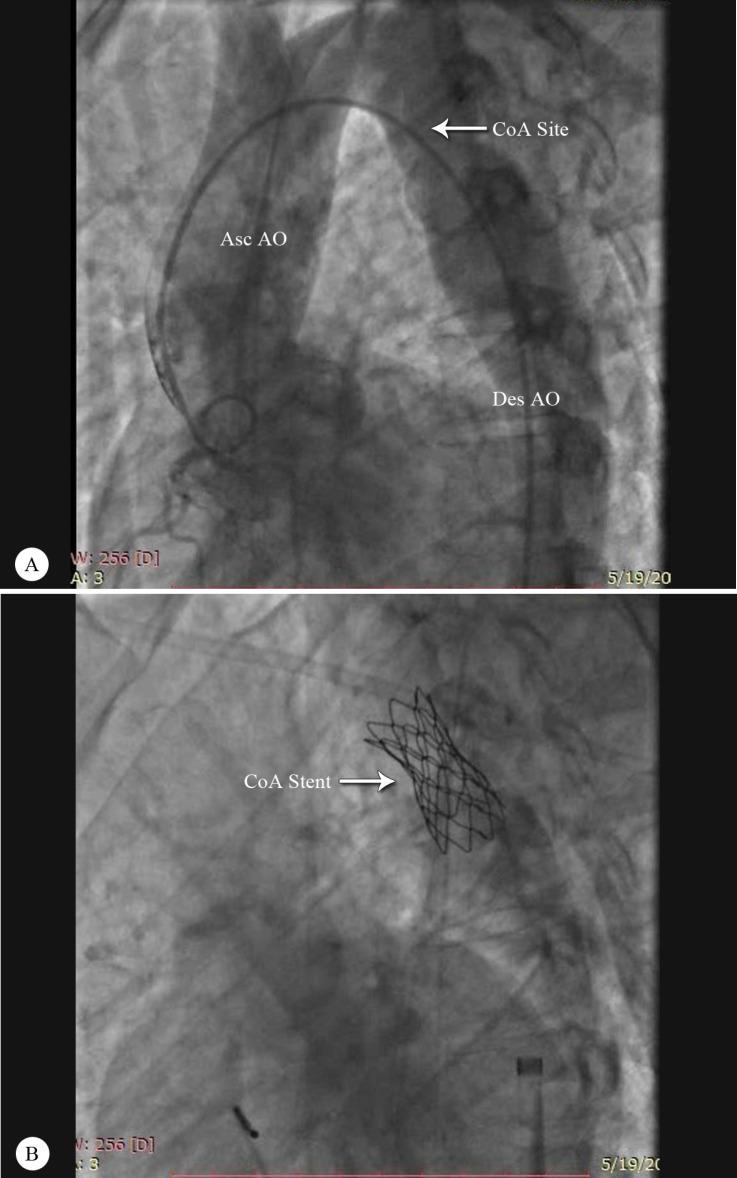
Aortic root angiogram in the left anterior oblique view, showing the following: A) Aortic coarctation is distal to the left subclavian artery. B) Percutaneous transluminal angioplasty is done successfully and the stent is seen in the proper position.

Valsartan (80 mg) and carvedilol (6.25 mg) were prescribed for the patient. Subsequently, for better evaluation, cardiac catheterization was done and its results confirmed the diagnosis of cc-TGA. Aortic root angiography demonstrated aortic coarctation distal to the emergence of the left subclavian artery (Figure 3-A). There was a gradient of around 80 mmHg across the coarctation, without any intracardiac shunting or significant valvular regurgitation.

With regard to the patient’s symptoms and the significant gradient of the coarctation site, transarterial coarctoplasty was done for him. After the wiring of the coarctation, a CP stent (Cheatham–Platinum stent) 8Z39 was deployed and postdilation was done using a Balloon-in-Balloon (BIB) (20×50 mm) with good results (gradient across the coarctation site=0) and without complications (Figure 3-B). The patient’s blood pressure was controlled with medical treatment, and echocardiography at the time of discharge and 6 months afterward showed the proper position of the aortic coarctation stent in the aorta, with a turbulent flow and a gradient of 25 mmHg without diastolic tail. The abdominal aorta had an antegrade diastolic flow.

CoA, Coarctation of the aorta; Asc Ao, Ascending aorta; Des AO, Descending aorta

## Discussion

Failure to identify the unusual position of the ventricles and the associated valves is a significant reason for cc-TGA to go undiagnosed in cardiology practice.^3^ The cardiac abnormalities associated with cc-TGA, systemic ventricular dysfunction, and arrhythmia development affect the clinical presentation and prognosis of patients.^4^ Related cardiovascular anomalies are reported in at least 90% of patients with cc-TGA.^5^ Although symptoms develop early in life, patients who lack intracardiac lesions do not present symptoms at that time. However, since such patients have a systemic right ventricle, they may appear with ventricular dysfunction and symptoms in their 40s or 50s.^6^

The most widely recognized cardiovascular lesions associated with cc-TGA are ventricular septal defects, which occur among 70%–80% of cases; left ventricular outflow tract obstructions, which are seen in 30%–60% of patients;^7^ and systemic AV anomalies, which are seen at autopsy in up to 90% of cases with cc-TGA.^5^ Regurgitation is frequent and generally progressive. An Ebstein-like malformation of the tricuspid valve, which is usually accompanied with right ventricular dysfunction and failure, has been reported in 20%–53% of patients. Mitral valve abnormalities occur in up to 50% of all cases. Tricuspid regurgitation is significantly related to mortality in both children and adults.^8^ There are some reports of the association between persistent ﬁfth aortic arch and coarctation of the aorta but not with cc-TGA.^9^ The combined hemodynamic impact of an inappropriate ventricle on the left side, aortic stenosis, and the Ebstenoid malformation of the left AV valve is fatal and aortic valve obstruction relief promotes AV regurgitation.^5^

This case had some interesting features. Firstly, the lack of intracardiac shunts. Allwork et al^10^ reported that only 15% of their patients with cc-TGA lacked cardiac shunts. Secondly, the case reported here is uncommon. Allwork et al,^10^ who studied 32 cases, and Schiebler et al,^11^ who reported a series of 33 autopsies, did not detect any aortic coarctation. Aida Brando et al^7^ reported 1 patient with cc-TGA and coarctation of the aorta without other associated anomalies; however, in our case, we found bicuspid aortic valve, which had not been reported previously. Different approaches are available for the treatment of cardiac anomalies, but endovascular treatment offers many advantages in the management of the stenosis of the great arteries.^12^

## Conclusion

In patients with a systemic right ventricle, the interventional procedure has been demonstrated to be effective for related symptomatic lesions or where critical hemodynamic advantage is foreseen. For the situation reported here, the interventional procedure was inevitable given that the obstruction of the aortic stream caused by aortic coarctation was delivering pressure over-burden on the fundamental right ventricle, which would lead to an early decay in its systolic capacity. We would advise careful physical examinations and complementary examinations before any therapeutic intervention.

## Notes:

***This paper should be cited as: ***Khajali Z, Maleki M, Mohebbi B, Aliramezany M. Coarctation Stenting in a Rare Case with Congenitally Corrected Transposition of the Great Arteries and the Bicuspid Aortic Valve. J Teh Univ Heart Ctr 2020;15(1):27-30.

## References

[1] Kosmas N, Nagueh SF, Kotileas P, Lachanis S, Xenakis K, Maragiannis D (2018). A rare combination of congenitally corrected transposition of the great arteries with pulmonary artery aneurysm. CASE (Phila).

[2] Wallis GA, Debich-Spicer D, Anderson RH (2011). Congenitally corrected transposition. Orphanet J Rare Dis.

[3] Bullock-Palmer RP, Rohen A (2009). Congenitally corrected transposition of the great arteries (CCTGA) initially presenting in the sixth decade. Echocardiography.

[4] Kayrak M, Kaya Z, Gul EE, Ulgen MS, Yazici M, Gumus S, Paksoy Y (2010). Congenitally corrected transposition of great arteries with severe rhythm disturbances. Indian Pacing Electrophysiol J.

[5] Patnaik AN, Barik R, Nemani L, Saikh S (2014). Aortic balloon valvuloplasty in corrected transposition of the great arteries with severe valvular aortic stenosis of congenital bicuspid aortic valve. Ann Pediatr Cardiol.

[6] Brida M, Diller GP, Gatzoulis MA (2018). Systemic right ventricle in adults with congenital heart disease: anatomic and phenotypic spectrum and current approach to management. Circulation.

[7] Brandão A, Magalhães S, Correia A (2004). Congenitally corrected transposition of the great arteries and aortic coarctation--an uncommon association. Rev Port Cardiol.

[8] Murtuza B, Barron DJ, Stumper O, Stickley J, Eaton D, Jones TJ, Brawn WJ (2011). Anatomic repair for congenitally corrected transposition of the great arteries: a single-institution 19-year experience. J Thorac Cardiovasc Surg.

[9] Khajali Z, Ali Basiri H, Maleki M (2011). Persistent fifth aortic arch associated with coarctation of aorta: a case report. Congenit Heart Dis.

[10] Allwork SP, Bentall HH, Becker AE, Cameron H, Gerlis LM, Wilkinson JL, Anderson RH (1976). Congenitally corrected transposition of the great arteries: morphologic study of 32 cases. Am J Cardiol.

[11] Schiebler GL, Edwards JE, Burchell HB, Dushane JW, Ongley PA, Wood EH (1961). Congenital corrected transposition of the great vessels: a study of 33 cases. Pediatrics.

[12] Khajali Z, Sanati HR, Pouraliakbar H, Mohebbi B, Aeinfar K, Zolfaghari R (2017). Self-expandable stent for repairing coarctation of the left-circumferential aortic arch with right-sided descending aorta and aberrant right subclavian artery with Kommerell's aneurysm. Ann Vasc Surg.

